# Sugar Sweetened Beverage Consumption Among Adults With Children in the Home

**DOI:** 10.3389/fnut.2018.00034

**Published:** 2018-05-04

**Authors:** Ashley H. White, Shirley A. James, Sjonna W. Paulson, Laura A. Beebe

**Affiliations:** ^1^Department of Biostatistics and Epidemiology, College of Public Health, University of Oklahoma Health Sciences Center, Oklahoma City, OK, United States; ^2^Oklahoma Tobacco Settlement Endowment Trust, Oklahoma City, OK, United States

**Keywords:** sugar-sweetened beverages, obesity, healthy diets, caregiver role in diet, soda consumption

## Abstract

**Introduction:** Consumption of sugar sweetened beverages (SSB)s has been linked with adult and childhood obesity, an increasing health burden in the United States. The aim of this study was to examine factors associated with the consumption of SSBs among Oklahoma adults with children in the home.

**Methods:** A random sample of 1,118 Oklahoma adults with children in the home participated in a survey about their SSB consumption between August and October, 2015. We calculated weighted prevalence estimates and examined the relationship between types of SSBs consumed and covariates of interest using logistic regression techniques appropriate for survey data. Outcome variables included three categories of SSB consumption: consuming ≥1 sugar-sweetened sodas daily, consuming ≥1 other SSBs daily, and total daily SSB consumption, defined as ≥1 SSB of any kind. Heavy consumers were those who drank ≥3 SSBs per day.

**Results:** Almost half (44%) of adults with children in the home consumed ≥1 total SSBs daily; 29% consumed ≥1 sugar-sweetened sodas and 28% consumed ≥1 other SSBs not including soda daily. The odds of consuming ≥1 SSBs daily was four times higher among those with a high school education or less (AOR = 4.06, 95% *CI* = 2.34, 7.04); almost three times higher for those who perceived their diet as somewhat healthy, or not very healthy (AOR = 2.72, 95% *CI* = 1.27, 5.82), more than double among those aged 18–34 years (AOR = 2.41, 95% *CI* = 1.08, 5.40), and almost double among those who consume <8 cups of water daily (AOR = 1.78, 95% *CI* = 1.06, 2.99).

**Conclusion:** Because SSBs have been linked with obesity, understanding factors associated with consumption is important, especially among parents and caregivers of children. These findings have implications for developing and targeting messages to prevent SSB consumption among those most at risk.

## Introduction

While recent data suggest that the rates of obesity are beginning to level, they remain at an alarmingly high rate (1). Using Behavioral Risk Factor Surveillance System data from 2016, Segal and associates reported adult obesity rates exceeded 30% in 25 states, with the highest occurring in West Virginia (37.7%) and with Oklahoma ranking ninth highest (32.8%) (1, 2). When combining adult overweight and obesity categories, the lowest rate was 58.1% (Colorado) and the highest rate was 71.3% (Mississippi) (2). Obesity disproportionately affects minority groups and those with low socioeconomic status (3–5). A 2013-14 National Health and Nutrition Examination Survey (NHANES) documented a significantly higher proportion of black Americans (48.4%) and Hispanic Americans (42.3%) were obese compared to their White counterparts (36.4%) (6). Additionally, analysis of 2014 BRFSS data revealed American Indian/Alaska Native adults had obesity rates ranging from 60.9% (North Carolina) to 93.9% (Ohio) (7).

Perhaps most alarming are trends among children which indicate 8.6% of Asian children, 14.7% of White children, 19.5% of African American children, and 21.9% of Hispanic children are obese (8). One factor, empty calories from discretionary foods like sugar-sweetened beverages (SSB), can contribute to obesity (9–13). which is associated with type 2 diabetes, heart disease, kidney disease, liver disease, tooth decay, and cancer, as well as other medical and mental health diagnoses (9–11, 14, 15). Decisions made by caregivers of children in the home impact not only short-term food and beverage choices, but long-term obesity (16–19).

SSBs are “any liquids sweetened with various forms of added sugars including brown sugar, corn sweetener, corn syrup, dextrose, fructose, glucose, high-fructose corn syrup, honey, lactose, malt syrup, maltose, molasses, raw sugar, and sucrose (20).” Examples of SSBs include regular soda (not sugar-free), fruit drinks, sports drinks, energy drinks, sweetened waters, and coffee and tea beverages with added sugars. Intake of SSBs is positively associated with increased body weight and risk of obesity, and negatively associated with the intake of important micronutrients (21).

While information on factors associated with adult SSB consumption is limited, surveillance studies have reported frequent adult consumers of SSBs are more likely to be younger (21–23), of African-American race or Hispanic ethnicity (21–23), males (22, 23), and those with lower socioeconomic status (21–23). In 2013, 66.4% of Oklahomans ages 18–24 years consumed one or more SSB daily, the highest prevalence of all states reporting (22). Additional research has uncovered associations with lower levels of education (21–23), unemployment (23), being physically inactive (23), smoking (23), and poor dietary habits (22, 23). Most research has focused on SSB consumption among adults and children, and not specifically among parents and caregivers of children.

In 2016, the Oklahoma Tobacco Settlement Endowment Trust launched “Shape Your Future: Rethink Your Drink,” an obesity prevention health communications program aimed at Oklahoma parents and caregivers of children. The program developed and released messages designed to combat SSB consumption by urging Oklahomans to “Rethink your Drink,” replacing SSBs with water. The “Rethink your Drink” campaign and messages originated from the Nutrition Education and Obesity Prevention Branch at the California Department of Public Health (24, 25). As part of the evaluation of the campaign, a series of cross-sectional surveys gathered information about Oklahomans' knowledge, attitudes, and behaviors concerning physical activity, nutrition, and overall wellness. Results presented here represent baseline SSB consumption in Oklahoma in 2015 prior to the launch of the “Rethink Your Drink” health communications campaign. The objective of this analysis was to examine factors associated with consumption of SSBs among Oklahoma adults with children in the home and to explore disparities in frequent consumption. Information about SSB consumption among parents and caregivers can be used to target messages to groups at highest risk. Given the critical role that parents and caregivers play in children's health behaviors, understanding these patterns may also influence the development and targeting of interventions for the prevention of childhood SSB consumption. Ongoing analysis of surveillance data is also important in detecting changes in obesity and consumption of SSBs on a national scale (11).

## Data and methods

The purpose of this telephone-based cross-sectional survey was to provide baseline data on adult consumption of SSBs prior to the launch of the statewide media campaign, “Shape Your Future: Rethink Your Drink.” Data collection occurred August through October, 2015 by the Sooner Survey Center in the University of Oklahoma College of Public Health. The sample population was a random sample of all non-institutionalized adults in Oklahoma with at least one child under the age of 18 living in the household and with either a cell or landline telephone. A target sample size of 1,000 completed surveys was determined to be sufficient for reliable estimates for subgroups of interest around key variables. Data were weighted to adjust for non-coverage and non-response, creating estimates more representative of the Oklahoma adult population with children living in the home. The survey instrument and protocol were approved by the University of Oklahoma Health Sciences Center Institutional Review Board.

### SSB survey questions

We measured SSB intake in our survey using two questions from the Behavioral Risk Factor Surveillance System sugary beverage module (26). Respondents were first asked: *During the past 30 days, how often did you drink regular soda or pop that contains sugar (do not include diet soda or diet pop)?* Respondents were then asked: *During the past 30 days, how often did you drink sugar-sweetened fruit drinks, sweet tea, sports drinks, or energy drinks?* Respondents were asked to NOT include 100% fruit juice, diet drinks, or artificially sweetened drinks. For both questions, respondents could answer in times per day, week, or month. Responses were converted to daily intake.

### SSB outcomes

Daily SSB consumption was examined for soda and other SSBs separately and in combination. This resulted in three outcomes, soda, other SSB, and all SSB, categorized as < 1 and 1 or more daily. As a secondary outcome, we created a category of heavy SSB consumers, those who drank three or more SSB per day.

### Covariates

Covariates included gender, three categories of age (<35, 35–54, and ≥55 years), four categories of race (White, American Indian/Alaska Native, African American and “other”), and two levels of education (high school degree or less and some college /technical school or more). Self-reported height and weight were used to calculate body mass index (BMI) (27). Two categories of BMI were included in the analysis, BMI <25 (underweight or normal), and BMI ≥25 (overweight or obese). Two self-assessed levels of perceived general health (excellent, very good, or good, vs. fair or poor), and two categories of perceived healthiness of diet (very healthy vs. somewhat, a little, or not at all healthy) were created, along with two categories of daily water consumption (8 or more cups or 0–7 cups). Finally the number of meals eaten at restaurants or fast food establishments was categorized as >2 times/week or ≤ 2 times/week.

### Data analysis

All analyses were performed using survey procedures in SAS 9.4. SSB prevalence estimates are presented as percentages with 95% confidence intervals. Chi-square tests were used to examine whether SSB consumption varied by the covariates of interest. *P* < 0.05 were considered statistically significant. Separate multiple logistic regression analyses were performed for soda, other SSB, and all SSB to model relationships between SSB consumption and the covariates described above. Variables in the model were removed using a manual stepwise method, with decisions based on the t-statistics of their estimated coefficients, using the associated *p*-values. Interaction between covariates was tested for significance. Adjusted odds ratios (aORS) and 95% CIs are reported. Finally, we analyzed heavy consumption, three or more SSB of any kind per day, to estimate associations with the covariates of interest.

## Results

1,118 Oklahomans with children in the home completed the survey using landline (*n* = 827) and cellular (*n* = 291) telephone numbers. The American Association for Public Opinion Research (AAPOR) response rate (RR1) for the survey was 10% and the cooperation rate was 90%, both in the acceptable range (28, 29). Participant characteristics are presented in Table 1. Respondents were predominantly female (54.1%) and white (76.6%). About half had a high school degree or less, and 60.9% were overweight or obese. The overall prevalence of ≥1 daily total SSB consumption was 44.1% (95% *CI* = 37.8, 50.4). Daily consumption of ≥1 sugar-sweetened sodas and other SSB was 28.8 and 28.4% respectively.

**Table 1:** Participant sociodemographic and health-related characteristics, and prevalence of daily SSB consumption by characteristic (% and 95% confidence interval).

**Characteristic**	**N (%^a^)**	**All SSB**	**SSB Sodas**	**Other SSB**
Total	1,118 (100)	44.1 (37.8, 50.4)	28.8 (22.5, 35.2)	28.4 (22.3, 34.6)
**GENDER**				
Male	348 (45.9)	44.3 (34.6, 54.0)	28.2 (18.9, 37.6)	33.5 (23.6, 43.4)
Female	767 (54.1)	43.4 (35.0, 51.8)	28.7 (19.9, 37.5)	24.4 (16.8, 32.0)
**AGE (YEARS)**				
18–34	180 (44.8)	48.5 (37.7, 59.4)	34.2, (22.9, 45.5)	31.8, (21.1, 42.6)
35–54	783 (46.4)	39.9 (32.0, 47.9)	24.6 (16.9, 32.2)	24.74 (17.0, 32.5)
≥55	155 (8.8)	43.4 (28.4, 58.3)	24.2 (10.3, 38.1)	31.0 (15.7, 45.7)
**RACE**				
White	883 (76.6)	45.9 (38.7, 53.1)	28.8 (21.3, 36.3)	30.5 (13.2, 47.7)
Black	66 (9.4)	28.1 (10.7, 45.5)	18.7 (3.1, 34.4)	16.4 (5.4, 27.4)
American Indian/Alaska Native	69 (9.2)	50.5 (32.4, 68.6)	35.0 (18.3, 51.7)	30.5 (13.2, 47.7)
Other	78 (4.8)	48.0 (12.1, 83.9)	46.2 (9.8, 82.6)	37.9 (0.0, 76.7)
**EDUCATION**				
≤ High school	184 (47.6)	59.2 (48.7, 69.7)^b^	40.4 (29.0, 51.8)^b^	38.9 (27.6, 50.1)^b^
>High school	915 (52.4)	30.9 (25.2, 36.7)	18.7 (13.7, 23.8)	19.4 (14.3, 24.4)
**BMI**				
Underweight/Normal (< 25)	469 (39.1)	42.4 (32.0, 52.8)	29.7 (19.5, 39.9)	26.6 (16.7, 36.5)
Overweight/Obese (≥25)	636 (60.9)	44.6 (36.5, 52.7)	27.4 (19.1, 35.6)	28.9 (21.0, 36.8)
**PERCEIVED HEALTH STATUS**				
Excellent, very good or good	973 (84.0)	42.4 (35.3, 49.5)	26.6 (19.4, 33.7)	27.7 (20.8, 34.7)
Fair or poor	143 (16.0)	50.8 (35.8 65.9)	38.2 (23.3, 53.1)	29.4 (16.1, 42.6)
**PERCEIVED HEALTHINESS OF DIET**				
Very healthy	245 (18.5)	21.6 (10.7, 32.5)^c^	12.4 (2.3, 22.5)^b^	12.1 (5.8, 18.4)^b^
Somewhat, a little or not at all healthy	870 (81.5)	48.9 (41.9, 56.0)	32.2 (24.8, 39.5)	31.8 (24.6, 39.0)
**DAILY WATER CONSUMPTION**				
≥8 cups	509 (50.7)	38.1 (28.6, 47.7)^c^	25.7 (16.3, 35.2)	21.3 (13.0, 29.6)^c^
0–7 cups	599 (49.3)	51.1 (42.8, 59.5)	32.9 (24.1, 41.6)	36.3 (27.3, 45.2)
**RESTAURANT/FAST FOOD MEALS**				
≤ 2 times/week	708 (66.7)	45.8 (37.8, 53.9)	30.2 (21.8, 38.7)	29.7 (21.8, 37.5)
>2 times/week	405 (33.3)	40.5 (30.4, 50.6)	25.4 (16.1, 34.6)	26.4 (16.1, 36.8)

a *Weighted percent*.

b *p < 0.001*.

c *p < 0.05*.

### Total SSB consumption

Almost 60% of those with a high school education or less reported drinking one or more SSB of any kind every day (59.2%), significantly higher than those with greater than a high school education (30.9%). The prevalence of daily total SSB consumption was also significantly higher among those who perceived their diets as less healthy (48.9 vs. 21.6%) and those who drink <8 cups of water daily (51.1 vs. 38.1%, Table 1). After adjusting for other variables in the model, the odds of consuming one or more SSBs daily was four times higher among those with a high school education or less (aOR = 4.06, 95% *CI* = 2.34, 7.04), and almost three times higher for those who perceive their diet as somewhat healthy, not very healthy, or not at all healthy (aOR = 2.72 with 95% *CI* = 1.27, 5.82). The odds of daily total SSB consumption was 2.41 times higher among those 18–34 years (95% *CI* = 1.08, 5.40), and almost double among those who consume <8 cups of water every day (aOR = 1.78, 95% *CI* = 1.06, 2.99, Table 2).

**Table 2:** Association between daily SSB consumption and sociodemographic and health-related characteristics (adjusted ORs and 95% confidence interval).

**Characteristic**	All SSB^*^	SSB Sodas^*^	Other SSB^*^
**GENDER**			
Female	(ref)	(ref)	(ref)
Male	1.75 (1.01, 3.02)	1.28 (0.62, 2.64)	2.66 (1.39, 5.06)^b^
**AGE (YEARS)**			
18–34	2.41 (1.08, 5.40)^b^	3.01 (1.07, 8.50)^b^	1.97 (0.75, 5.22)
35–54	1.31 (0.61, 2.79)	1.62 (0.64, 4.10)	1.13 (0.47, 2.70)
≥55	(ref)	(ref)	(ref)
**RACE**			
White	(ref)	(ref)	(ref)
Black	0.32 (0.10, 1.06)	0.41 (0.10, 1.71)	0.39 (0.13, 1.11)
American Indian/Alaska Native	0.93 (0.37, 2.38)	1.04 (0.36, 3.04)	1.08 (0.44, 2.66)
Other	1.13 (0.31, 4.05)	2.60 (0.69, 9.86)	1.63 (0.35, 7.66)
**EDUCATION**			
≤ High school	4.06 (2.34, 7.04)^a^	3.25 (1.62, 6.52)^a^	3.13 (1.63, 6.03)^a^
>High school	(ref)	(ref)	(ref)
**BMI**			
Underweight/Normal (< 25)	(ref)	(ref)	(ref)
Overweight/Obese (≥25)	0.89 (0.48, 1.64)	0.76 (0.0.36, 1.63)	0.98 (0.49, 1.97)
**PERCEIVED HEALTH STATUS**			
Fair or poor	1.09 (0.49, 2.42)	1.46 (0.66, 3.21)	0.93 (0.41, 2.10)
Excellent, very good or good	(ref)	(ref)	(ref)
**PERCEIVED HEALTHINESS OF DIET**			
Very healthy	(ref)	(ref)	(ref)
Somewhat, a little or not at all healthy	2.72 (1.27, 5.82)^b^	2.39 (0.84, 6.80)	2.81 (1.26, 6.27)^b^
**DAILY WATER CONSUMPTION**			
≥8 cups	(ref)	(ref)	(ref)
0–7 cups	1.78 (1.06, 2.99)^b^	1.32 (0.66, 2.65)	2.66 (1.45, 4.90)^b^
**RESTAURANT/FAST FOOD MEALS**			
≤ 2 times/week	(ref)	(ref)	(ref)
>2 times/week	0.79 (0.46, 1.35)	0.84 (0.44, 1.60)	0.82 (0.44, 1.55)

* *Adjusted for gender, age, race, education, BMI, perceived health status, perceived healthiness of diet, daily water consumption and meals eaten outside the home*.

a *p < 0.001*.

b *p < 0.05*.

### Sugar sweetened soda consumption

The prevalence of sugar-sweetened soda consumption was significantly higher among those with lower levels of education (40.4 vs. 18.7%) and those who perceive their diets as less healthy (32.2 vs. 12.4%, Table 1). After adjusting for other variables in the model, the odds of consuming one or more sugar-sweetened soda per day was three times higher among those with a high school education or less (aOR = 3.25, 95% *CI* = 1.62, 6.52), and three times higher among those aged 18–34 years (aOR = 3.01, 95% *CI* = 1.07, 8.50, Table 2).

### SSB consumption not including soda

Similar to soda consumption, prevalence of other SSB consumption was significantly higher among those with lower levels of education (38.9 vs. 19.4%) and those who perceive their diets as less healthy (31.8 vs. 12.1%). Additionally, the prevalence was higher among those reporting <8 cups of water per day (36.3 vs. 21.3%, Table 1). After adjusting for other variables in the model, the odds of consuming one or more SSB excluding soda per day was three times greater among those with a high school education or less (aOR = 3.13, 95% *CI* = 1.63, 6.03), almost three times greater for those who perceive their diet as somewhat healthy, not very healthy, or not at all healthy (aOR = 2.81, 95% *CI* = 1.26, 6.27), and more than double among those consume <8 cups of water every day (aOR = 2.66, 95% *CI* = 1.45, 4.90). The odds of daily SSBs other than soda was 2.66 times higher among males as compared to females (95% *CI* = 1.39, 5.06, Table 2).

### Heavy total SSB consumption

Results were similar for heavy SSB consumption, defined as consuming three or more SSBs of any kind per day. The prevalence was highest among those with lower levels of education (27.1 vs. 7.0%), those who perceive their diet as less healthy (18.4 vs. 5.3%), and those who drink <8 cups of water each day (23.6 vs. 9.8%, Table 3). Although those with fair or poor perception of general health had the highest prevalence of heavy SSB consumption (30.2%), the association between perceived health and heavy consumption did not remain after adjusting for other covariates in the model. After adjusting for other variables in the model, the odds of consuming three or more SSBs daily was seven times higher among those with a high school education or less (aOR = 7.15, 95% *CI* = 2.86, 17.91), six times higher among those aged 18–34 years (aOR = 6.08, 95% *CI* = 1.45, 25.59), and four times higher among those who consume <8 cups of water every day (aOR = 4.74, 95% *CI* = 1.91, 11.73, Table 3).

**Table 3:** Association between heavy SSB (3 or more per day) consumption and sociodemographic and health-related characteristics (adjusted ORs and 95% confidence interval).

**Characteristic**	**Heavy SSB N (%^a^)**	aOR (95% CI)^*^
Total	86 (16.4)	
**GENDER**		
Male	33 (17.1)	2.12 (0.86, 5.23)
Female	51 (14.9)	(ref)
**AGE (YEARS)**		
18–34	23 (18.1)	6.08 (1.45, 25.59)
35–54	52 (15.2)	2.75 (0.80, 9.48)
≥55	11 (13.7)	(ref)
**RACE**		
White	62 (15.7)	(ref)
Black	6 (7.7)	0.27 (0.05, 1.37)
American Indian/Alaska Native	12 (24.8)	1.15 (0.37, 3.61)
Other	6 (33.9)	4.82 (0.79, 29.34)
**EDUCATION**		
≤ High school	37 (27.1)^b^	7.15 (2.86, 17.91)
> High school	49 (7.0)	(ref)
**BMI**		
Underweight/Normal (25)	30 (16.7)	(ref)
Overweight/Obese (≥25)	55 (16.7)	1.03 (0.38, 2.80)
**PERCEIVED HEALTH STATUS**		
Excellent, very good or good	56 (13.1)^c^	(ref)
Fair or poor	28 (30.2)	2.42 (0.94, 6.25)
**PERCEIVED HEALTHINESS OF DIET**		
Very healthy	8 (5.3)^c^	(ref)
Somewhat, a little, or not at all healthy	77 (18.4)	2.05 (0.60, 7.00)
**DAILY WATER CONSUMPTION**		
≥ 8 cups	23 (9.8)^c^	(ref)
0–7 cups	63 (23.6)	4.74 (1.91, 11.73)
**RESTAURANT/FAST FOOD MEALS**		
≤ 2 times/week	47 (16.4)	(ref)
>2 times/week	36 (16.1)	1.07 (0.46, 2.50)

* *Adjusted for gender, age, race, education, BMI, perceived health status, perceived healthiness of diet, daily water consumption and meals eaten outside the home*.

a *Weighted percent*.

b *p < 0.001*.

c *p < 0.05*.

## Discussion

The results of this study indicate that in 2015, 44.1% of Oklahomans with children in the home consumed one or more SSBs every day. Daily consumption of sugar-sweetened soda and other SSBs was similar, 28.8 and 28.4%, respectively. In 2013, Oklahoma was one of six states with the highest consumption of SSBs (22) with an adjusted prevalence of 44.6% of Oklahoma adults consuming ≥1 SSB daily (2). While the association between obesity and low SES, low educational levels, and racial differences is well documented in the literature, the association between these variables and the consumption of SSB is less well-known, especially among adults with children in the home.

Consistent with prior studies, daily SSB consumption in our sample of adults was associated with lower levels of education (21–23), male gender (22, 23), and younger age for heavy consumption (21–23). Perceived less healthy diet and drinking <8 cups of water per day were also consistently associated with SSB in our study. Tasevska and associates also reported an association between consumption of SSB and eating a healthy diet (measured by eating breakfast 6 or 7 days per week) (30). Although Qobadi and Payton reported an association between consumption of SSBs and eating in fast-food restaurants, our study did not uncover a similar association (23).

In this study of Oklahoma adults with children in the home, education was the factor most consistently associated with SSB consumption. The odds ratios (OR) were robust and showed an inverse association between education and consumption of SSBs. Han and Powell reported similar, but less robust results in their study on the association between lower levels of education and consumption of total SSBs (*OR* = 1.34, 95% *CI* = 1.23, 1.47) or sodas only (*OR* = 1.52, 95% *CI* = 1.39, 1.67) (21), as did Qobadi and Payton, in their study of SSB consumption in Mississippi (*OR* = 1.9, 95% *CI* = 1.4, 2.6) (23). Two additional studies revealed an association between choosing SSBs for children and lower levels of education (30, 31). Most striking in our study was the association between education and consumption of three or more SSBs per day. Those with a high school education or less were four times more likely to consume three or more SSB daily than those with higher educational attainment. One reason might be referenced by Han and Powell, who noted that lower income households may choose lower cost SSBs over more expensive, but healthier alternatives like milk or non-sweetened fruit juices (21). Beverage choices parents make can influence their children's choices both now and in the future (12). Given the consistent findings related to lower educational attainment and higher consumption of SSBs, limited knowledge about SSBs and their calorie content could be a factor influencing high prevalence. For a woman on a 1,800 calorie diet, one 12 ounce serving of a sugar- sweetened beverage represents about 140 calories or 8% of the total recommended daily caloric intake from empty calories, while for a man on a 2,000 calorie diet, a typical serving of a SSB accounts for 7% of daily calories. This modifiable risk factor can be addressed with educational campaigns specifically targeting low SES families (1).

A novel finding in our study was the association between drinking less than the recommended level of eight cups of water daily and consumption of SSBs (AOR = 1.78, 95% *CI* = 1.06, 2.99). People often consider dietary changes when attempting to lose weight (25). This study suggests that messages promoting a higher level of water intake on a daily basis could decrease SSB consumption, affecting caloric intake. This study provides further evidence for the media message, “Rethink your drink (25).”

The findings from this study are somewhat unique as they reflect adults with children in the home, meaning parents and caregivers of children. The population-based sample is weighted to reflect the population of adults with children in the home in Oklahoma, and may be generalizable to other states with similar demographics. Our findings related to heavy consumption of SSBs are also somewhat novel. However, this study also has some notable limitations. First, the consumption of SSBs is only one of many prevalent obesogenic behaviors. Our study is limited in its focus on SSB consumption and further limited to adults with children in the household. Consumption of SSB among the children in the household was not measured in our survey (30). Second, cross-sectional studies have prediction limitations. Because outcome and covariates are assessed at the same point in time, no causal assumptions can be made. Further, as with any survey study, the data represented here are prone to bias, particularly recall bias.

## Conclusions

This study indicates that lower educational levels, perceived healthiness of diet, drinking <8 cups of water daily, male gender, and younger age were associated with daily consumption of SSBs among adults with children in the home. Sugary drinks are a major source of excess sugar and calories in the diet (9). Reducing SSB consumption among adults and children is a priority given its association with rising levels of obesity. Health communication and mass media campaigns are considered an evidence-based practice for improving knowledge, attitudes, and behaviors. The Rethink Your Drink campaign and similar campaigns have shown promise in other states in reducing consumption of SSBs (25, 32, 33). Given the high rate of SSB consumption among adults with children in the home demonstrated in this study, such a targeted intervention is needed to reduce the risk of being overweight and obese (34).

## Author contributions

AW serves as Director of the Sooner Survey Center in the University of Oklahoma College of Public Health. She was responsible for all data collection and compilation. SJ was responsible for data analysis and authoring the research paper. SP was instrumental in defining the research questions, decisions around data collection, and interpretation of the data. LB supervised the research project from its initial planning, and contributed to question development, data collection, data analysis, and data interpretation.

### Conflict of interest statement

The authors declare that the research was conducted in the absence of any commercial or financial relationships that could be construed as a potential conflict of interest.

## References

[1] Segal LM Rayburn J Beck SE The state of obesity: better policies for a Healthier America. Princeton, NJ: Robert Wood Johnson Foundation, Trust for America's Health (2017). Available online at: http://healthyamericans.org/assets/files/TFAH-2017-ObesityReport-FINAL.pdf

[2] Behavioral Risk Factor Surveillance System Survey Data Documetation. Centers for DIsease Control Prevention (2016). Available online at: https://www.cdc.gov/brfss/index.html

[3] Lebel AKY Clary C Bisset S Subramanian SV. Geographic variability in the association between socioeconomic status and BMI in the USA and Canada. PLoS ONE (2014) 9:e99158. 10.1371/journal.pone.0099158 24932774 PMC4059636

[4] Wang LSJ Wang K Bailey BA Alamian A Stevens MA Wang Y. Ethnic differences in risk factors for obesity among adults in California, the United States. J Obes. (2017) 2017:2427483. 10.1155/2017/2427483 28352473 PMC5352906

[5] Ogden CL Carroll MD Kit BK Flegal KM. Prevalence of childhood and adult obesity in the United States, 2011-2012. Jama (2014) 311:806–14. 10.1001/jama.2014.732 24570244 PMC4770258

[6] Ogden CL Fryar CD FLegal KM Prevalence of obestiy among adults and youth: United States, 2011–2014. 26633046

[7] Kaiser F Overweight and obesity rates for adults by race/Ethnicity. In: Foundation K. Available online at: https://www.kff.org/other/state-indicator/adult-overweightobesity-rate-by-re/?currentTimeframe=0&sortModel=%7B%22colId%22:%22Location%22,%22sort%22:%22asc%22%7D (2016).

[8] Flegal KM Kruszon-Moran D Carroll MD Fryar CD Ogden CL. Trends in obesity among adults in the United States, 2005 to 2014. Jama (2016) 315:2284–91. 10.1001/jama.2016.6458 27272580 PMC11197437

[9] Malik VS Schulze MB Hu FB. Intake of sugar-sweetened beverages and weight gain: a systematic review. Am J Clin Nutr. (2006) 84:274–88. 10.1093/ajcn/84.2.274 16895873 PMC3210834

[10] Malik VS Popkin BM Bray GA Després J-P Hu FB. Sugar sweetened beverages, obesity, type 2 diabetes and cardiovascular disease risk. Circulation (2010) 121:1356–64. 10.1161/CIRCULATIONAHA.109.876185 20308626 PMC2862465

[11] Malik VS Pan A Willett WC Hu FB. Sugar-sweetened beverages and weight gain in children and adults: a systematic review and meta-analysis. Am J Clin Nutr. (2013) 98:1084–1102. 10.3945/ajcn.113.058362 23966427 PMC3778861

[12] Schulze MB Manson JE Ludwig DS Colditz GA Stampfer MJ Willett WC. Sugar-sweetened beverages, weight gain, and incidence of type 2 diabetes in young and middle-aged women. Jama (2004) 292:927–34. 10.1001/jama.292.8.927 15328324

[13] Palmer JR Boggs DA Krishnan S Hu FB Singer M Rosenberg L. Sugar-sweetened beverages and incidence of type 2 diabetes mellitus in African American women. Arch Intern Med. (2008) 168:1487–92. 10.1001/archinte.168.14.1487 18663160 PMC2708080

[14] Bomback AS Derebail VK Shoham DA Anderson CA Steffen LM Rosamond WD. Sugar-sweetened soda consumption, hyperuricemia, and kidney disease. Kidney Int. (2010) 77:609–16. 10.1038/ki.2009.500 20032963 PMC3299001

[15] Bernabe E Vehkalahti MM Sheiham A Aromaa A Suominen AL. Sugar-sweetened beverages and dental caries in adults: a 4-year prospective study. J Dent. (2014) 42:952–58. 10.1016/j.jdent.2014.04.011 24813370

[16] Yee AZH Lwin MO Ho SS. The influence of parental practices on child promotive and preventive food consumption behaviors: a systematic review and meta-analysis. Int J Behav Nutr Phys Act. (2017) 14:47. 10.1186/s12966-017-0501-3 28399881 PMC5387370

[17] Zahid A Davey C Reicks M. Beverage intake among children: associations with parent and home-related factors. Int J Environ Res Public Health (2017) 14:E929. 10.3390/ijerph14080929 28820455 PMC5580631

[18] Popkin BM. Patterns of beverage use across the lifecycle. Physiol Behav. (2010) 100:4–9. 10.1016/j.physbeh.2009.12.022 20045423 PMC2849916

[19] Mazarello Paes V Hesketh K O'Malley C Moore H Summerbell C Griffin S. Determinants of sugar-sweetened beverage consumption in young children: a systematic review. Obes Rev. (2015) 16:903–13. 10.1111/obr.12310 26252417 PMC4737242

[20] Prevention CfDCa Get the Facts: Sugar-Sweetened Beverages and Consumption. (2017).

[21] Han E Powell LM. Consumption patterns of sugar sweetened beverages in the United States. J Acad Nutr Diet. (2013) 113:43–53. 10.1016/j.jand.2012.09.016 23260723 PMC3662243

[22] Park S Xu F Town M Blanck HM. Prevalence of sugar-sweetened beverage intake among adults-23 states and the District of Columbia, 2013. Morb Mortal Wkly Rep. (2016) 65:169–174. 10.15585/mmwr.mm6507a1 26914018

[23] Qobadi M Payton M. Consumption of sugar-sweetened beverages in mississippi: is there a disparity? behavioral risk factor surveillance system, 2012. Int J Environ Res Public Health (2017) 14:228. 10.3390/ijerph14030228 28245580 PMC5369064

[24] Richardson J Brown K Rethink your Drink Campaign and Lessons (California). California Department of Health Nutrition education and Obesity Prevention Branch (2014). https://snaped.fns.usda.gov/materials/rethink-your-drink-campaign-lessons-california

[25] Boles M Adams A Gredler A Manhas S. Ability of a mass media campaign to influence knowledge, attitudes, and behaviors about sugary drinks and obesity. Prev Med. (2014) 67 (Suppl. 1):S40–5. 10.1016/j.ypmed.2014.07.023 25066020

[26] Park S Pan L A Data User's Guide to the BRFSS Sugar-Sweetened Beverage Questions: How to Analyze Consumption of Sugar-Sweetened Beverages. Atlanta, GA: Center for Disease control and Prevention.

[27] Brener ND McManus T Galuska DA Lowry R Wechsler H. Reliability and validity of self-reported height and weight among high school students. J Adolesc Health (2003) 32:281–87. 10.1016/S1054-139X(02)00708-5 12667732

[28] Keeter S Hatley N Kennedy C Lau A What Low Response Rates Mean for Telephone Surveys. Pew Research Center Publication (2017). Available online at: http://www.pewresearch.org/2017/05/15/what-low-response-rates-mean-for-telephone-surveys/

[29] The American Association for Public Opinion Research 2016 Standard Definitions: Final Dispositions of Case Codes and Outcome Rates for Surveys. 9th Edn AAPOR (2016).

[30] Tasevska N DeLia D Lorts C Yedidia M Ohri-Vachaspati P. Determinants of sugar-sweetened beverage consumption among low-income children: are there differences by race/ethnicity, age, and sex? J Acad Nutr Diet. (2017) 117:1900–20. 10.1016/j.jand.2017.03.013 28495478 PMC5677590

[31] Hennessy M Bleakley A Piotrowski JT Mallya G Jordan A. Sugar-sweetened beverage consumption by adult caregivers and their children: the role of drink features and advertising exposure. Health Educ Behav. (2015) 42:677–86. 10.1177/1090198115577379 25794520

[32] Barragan NC Noller AJ Robles B Gase LN Leighs MS Bogert S The “Sugar Pack” health marketing campaign in Los Angeles County, 2011-2012. Health Promot Pract. (2013) 15:208–16. 10.1177/1524839913507280 24149214

[33] Farley TA Halper HS Carlin AM Emmerson KM Foster KN Fertig AR. Mass media campaign to reduce consumption of sugar-sweetened beverages in a rural area of the United States. Am J Public Health (2017) 107:989–95. 10.2105/AJPH.2017.303750 28426298 PMC5425869

[34] Park J Lin HC Peng CY. The supplemental nutrition assistance program and frequency of sugar-sweetened soft drink consumption among low-income adults in the US. Nutr Health (2017) 23:147–57. 10.1177/0260106017726248 28820019

